# Evaluating prompt engineering on GPT-3.5’s performance in USMLE-style medical calculations and clinical scenarios generated by GPT-4

**DOI:** 10.1038/s41598-024-66933-x

**Published:** 2024-07-28

**Authors:** Dhavalkumar Patel, Ganesh Raut, Eyal Zimlichman, Satya Narayan Cheetirala, Girish N Nadkarni, Benjamin S. Glicksberg, Donald U. Apakama, Elijah J. Bell, Robert Freeman, Prem Timsina, Eyal Klang

**Affiliations:** 1 Mount Sinai Health System, New York, USA; 2 Hospital Management, Sheba Medical Center, Affiliated to Tel-Aviv University, Tel Aviv, Israel; 3 ARC Innovation Center, Sheba Medical Center, Affiliated to Tel-Aviv University, Tel Aviv, Israel; 4 The Charles Bronfman Institute of Personalized Medicine, Icahn School of Medicine at Mount Sinai, New York, NY USA; 5 University of California, Los Angeles, USA

**Keywords:** Large language models, Prompt engineering, GPT-3.5, GPT-4, Medical problem-solving, USMLE, Chain of thoughts, Modified chain of thoughts, Machine learning, Health care

## Abstract

This study was designed to assess how different prompt engineering techniques, specifically direct prompts, Chain of Thought (CoT), and a modified CoT approach, influence the ability of GPT-3.5 to answer clinical and calculation-based medical questions, particularly those styled like the USMLE Step 1 exams. To achieve this, we analyzed the responses of GPT-3.5 to two distinct sets of questions: a batch of 1000 questions generated by GPT-4, and another set comprising 95 real USMLE Step 1 questions. These questions spanned a range of medical calculations and clinical scenarios across various fields and difficulty levels. Our analysis revealed that there were no significant differences in the accuracy of GPT-3.5's responses when using direct prompts, CoT, or modified CoT methods. For instance, in the USMLE sample, the success rates were 61.7% for direct prompts, 62.8% for CoT, and 57.4% for modified CoT, with a p-value of 0.734. Similar trends were observed in the responses to GPT-4 generated questions, both clinical and calculation-based, with p-values above 0.05 indicating no significant difference between the prompt types. The conclusion drawn from this study is that the use of CoT prompt engineering does not significantly alter GPT-3.5's effectiveness in handling medical calculations or clinical scenario questions styled like those in USMLE exams. This finding is crucial as it suggests that performance of ChatGPT remains consistent regardless of whether a CoT technique is used instead of direct prompts. This consistency could be instrumental in simplifying the integration of AI tools like ChatGPT into medical education, enabling healthcare professionals to utilize these tools with ease, without the necessity for complex prompt engineering.

## Introduction

ChatGPT and other large language models (LLMs) are venturing into clinical and research areas^1–5^. Prompt engineering is considered to be key in enhancing the performance of these models^6,7^. Research is looking into its use in medical contexts, such as evaluating soft skills through USMLE questions^8–10^.

Recent data shows ChatGPT (GPT-3.5) scores about 60% on the USMLE. GPT-4 scores an even more impressive 87%^9,10^. Studies of GPT-4's ability to craft multi-choice medical tests show AI's potential role in education^11^. The “chain of thoughts” (CoT) prompt method is designed to encourage the LLM to “reason step by step”, thereby, mimicking human problem-solving methods. However, CoT didn’t significantly boost GPT-4's USMLE scores. This is notable because the USMLE relies minimally on math^12,13^. CoT has proven useful in many non-medical tasks^6,7^. However, its impact on USMLE medical questions, particularly those with calculations, is still uncertain.

Our research focuses on GPT-3.5's performance with USMLE-like questions, separated into clinical and calculation-focused questions. We tested direct prompts, CoT, and a modified CoT. We reviewed various prompting strategies, and then selected direct prompts, Chain of Thought (CoT), and a modified CoT for our study. The choice of these strategies was influenced by their extensive discussion and acknowledgment in recent research. We chose direct prompting for its straightforward method of posing questions without prior context, serving as a key benchmark to assess the model's capacity to formulate responses based on its existing knowledge. Following this, CoT was applied to determine whether simulating human problem-solving techniques could enhance clinical reasoning. We then introduced mCoT, which adds further guidance and constraints to the CoT method to more closely tailor the model's thought process to the intricate demands of medical questions. Our study included questions generated by GPT-4 and actual USMLE Step 1 exams.

## Methods

### Study design

**Figure 1:**
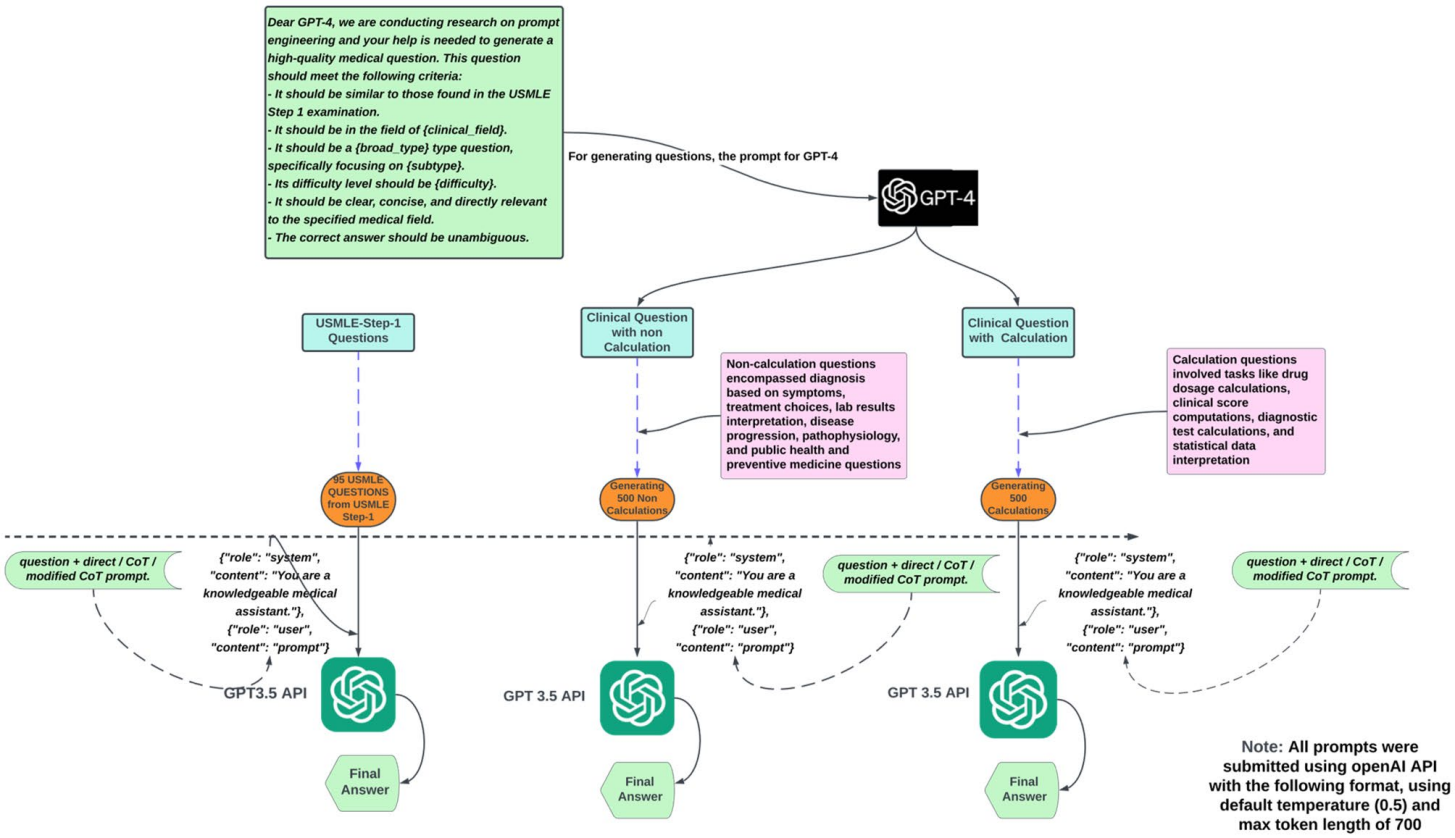
The figure illustrates a multi-step process in which GPT-4 generates 1000 USMLE-style medical questions with calculation and non-calculation, and GPT-3.5-turbo answers them using three prompting strategies direct, COT, and Modified COT. The generated questions span 19 clinical fields and various medical topics, and the model's answers aim to mimic human problem-solving behavior, enhancing reasoning ability and clarity in its responses.

We evaluated ChatGPT (GPT-3.5-turbo) using three prompt strategies: direct, chain of thoughts (CoT), and a modified CoT^14^. Our analysis covered 95 USMLE Step 1 multiple-choice questions^15^. We also added two sets of questions we created with GPT-4, one set of medical calculations and another of clinical case questions, and the architecture and workflow of this experiment are detailed in Fig. 1. Analyses were performed during July 2023.

### Question generation

We used GPT-4 to create 1000 questions in USMLE style^11,16^. We split them evenly into two groups: 500 calculation-based and 500 non-calculation-based. The non-calculation set spanned diagnoses, treatment plans, lab test readings, disease courses, pathophysiology, public health, and preventive care. The calculation set included tasks like figuring out medication doses, clinical scores, diagnostic math, and statistical evaluations. GPT-4 also rated each question by difficulty—easy, medium, or hard, and by the medical field, covering 19 specialties such as Internal Medicine, Pediatrics, Psychiatry, Surgery, and others.

For generating questions, the prompt was:

Dear GPT-4, we are conducting research on prompt engineering and your help is needed to generate a high-quality medical question. This question should meet the following criteria:

- It should be like those found in the USMLE Step 1 examination.
- It should be in the field of {clinical field}.

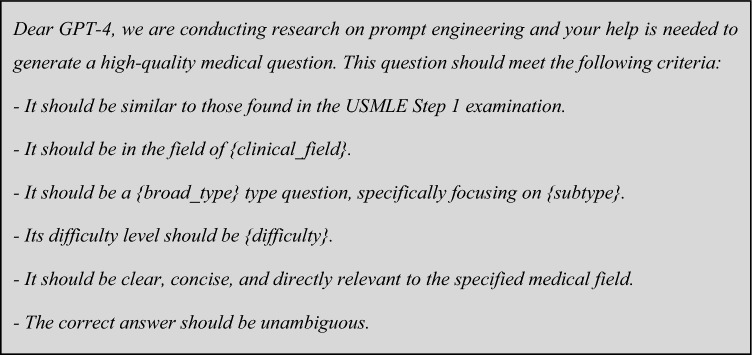


The result should be returned in a JSON format, with the following headers:
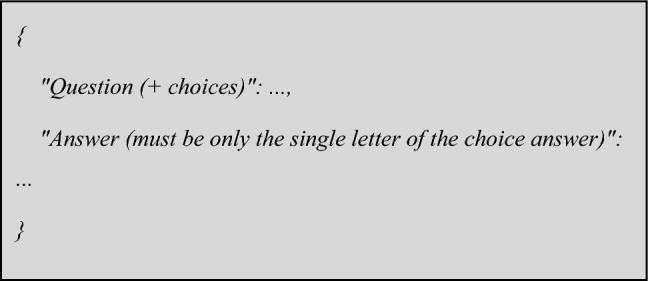


where

- *Clinical field* one of 19 clinical fields (internal medicine, surgery, etc.).
- *Broad type* either calculation or non-calculation.
- *Subtypes*:
- Calculation questions: Drug dosage calculations, Clinical score calculations, Diagnostic test calculations, Statistical data interpretation.
- Non-calculation type questions: Diagnosis based on symptoms, Treatment selection, Interpretation of lab results, Disease progression and prognosis, Pathophysiology questions, and public health and preventive medicine questions.
- *Difficulty level* easy, medium, or hard.

### Question answering—prompt engineering

To query GPT-3.5, we used three prompting strategies:

- The "direct prompt" strategy simply instructed the model to "answer the question."
- The "CoT" strategy guided the model to "reason step by step and answer the question."
- The "modified CoT" strategy directed the model to "read the problem carefully, break it down, devise a strategy for solving it, check each step for accuracy, and clearly and concisely convey your reasoning leading to your final answer." This approach sought to mimic human problem-solving behavior, with the aim to enhance the model's reasoning ability while promoting clarity and precision in its responses.

All prompts were submitted using openAI API with the following format, using default temperature (0.5) and max token length of 700:
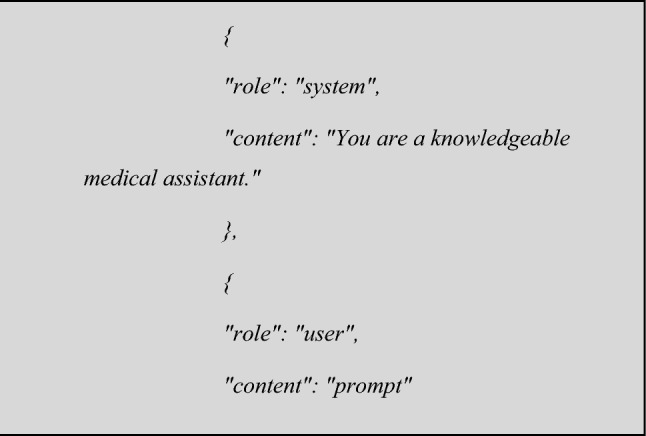


where *'prompt'* corresponded to:
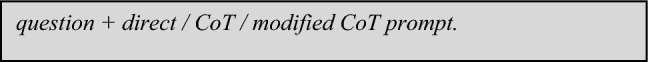


The direct prompt was:
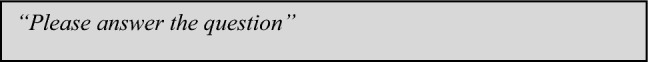


The CoT prompt was:
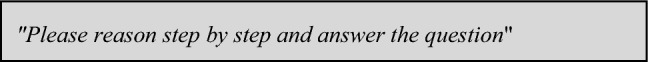


The modified CoT prompt was:
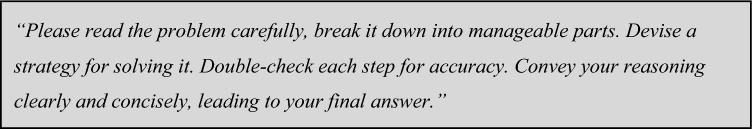


### Human validation

Two emergency room attending physicians independently evaluated the first 50 questions generated by GPT-4 for appropriateness, type, subtype, difficulty level, clinical field, and correctness of the answer. Each aspect was reviewed blindly, and the assessments were quantified.

To clearly delineate the agreement calculations, each evaluator's judgments were compared against the features of GPT-4 generated questions, for example, appropriateness, type, and difficulty level. The percentage agreement for each evaluator was calculated by the ratio of matches (e.g. agreement with GPT-4 difficulty level assignment) to the total questions evaluated. We further analyzed the inter-rater reliability between the evaluators using Cohen’s Kappa to compare their levels of agreement.

### Evaluation

The main metric of our evaluation was the accuracy of GPT-3.5 and GPT-4 answers and, we have mentioned their feature comparison in Table 1. In addition, we ran further analyses on the questions, looking at difficulty level, what type they were, and their medical specialty. This helped us get a full picture of ChatGPT's capabilities.

**Table 1:** Comparison of Features between GPT-3.5 and GPT-4.

Features	GPT-3	GPT-4
Performance	Outperformed its predecessors	Scoring 40% higher on internal factual performance benchmark
Model size	175 billion parameters	Estimated to have over 10 trillion parameters
Steerability	Capable of changing its behavior	Designed to be more steerable
Alignment	Not specifically designed for alignment improvement	Designed to improve model alignment, resulting in more truthful output
Image input	Can only use text input	Can use image inputs and text
Multilingual support	Supports multiple languages	Improved multilingual support compared to GPT-3
Training data	Trained on a large dataset but not as diverse as GPT-4	Trained on a larger dataset

### Statistical analysis

Statistical analyses were executed using Python version 3.9.16. Agreement between the human reviewers was statistically analyzed using Cohen’s Kappa to measure inter-rater reliability. We used the Chi-square test to examine the relation between prompt types and response accuracy. A p-value of less than 0.05 was considered statistically significant.

## Results

Table 2 presents the aggregated results of the human readers’ validations. It shows the percentage of agreement between the two reviewers across various categories and the Cohen's Kappa values, indicating the degree of inter-rater reliability. The evaluations revealed high agreement in most categories, with variability in the assessment of question difficulty.

**Table 2:** Two emergency room physicians' validations of GPT-4 generated USMLE questions and inter-rater agreement rates.

Review category	Human 1 agreement (percentage)	Human 2 agreement (percentage)	Cohen’s Kappa
Appropriateness of question	90%	90%	0.78
Appropriateness of type	100%	100%	1.00
Appropriateness of subtype	98%	98%	1.00
Agreement on difficulty level	58% (medium: 37.9%, hard: 37.9%, easy: 24.1%)	44% (medium: 36.4%, easy: 36.4%, hard: 27.3%)	0.41
Appropriateness of clinical field	90%	96%	1.00
Correctness of answer	76%	76%	0.78

Our study's main findings are presented in Table 3. We checked GPT-3.5's performance with three prompt types: direct, chain of thoughts (CoT), and modified CoT. We tested this performance across three question sets: USMLE Step 1 samples, GPT-4 generated clinical questions, and GPT-4 generated calculation questions.

**Table 3:** Comparison of the effectiveness of three prompt methods with ChatGPT for USMLE Step 1 samples, clinical non-calculation questions from GPT-4, and calculation questions from GPT-4 (Chi-square statistic was used to calculate P value).

	ChatGPT direct prompt	ChatGPT CoT prompt	ChatGPT Modified CoT prompt	P value
USMLE step 1 sample	54/95 (61.7%)	59/95 (62.8%)	58/95 (57.4%)	0.734
GPT-4 clinical questions	270/500 (54.0%)	274/500 (54.8%)	257/500 (51.4%)	0.530
GPT-4 calculation questions	397/500 (79.4%)	398/500 (79.6%)	386/500 (77.2%)	0.589

Across calculation, clinical, and USMLE Step 1 questions, the different prompting methods—direct, CoT, and modified CoT—showed no significant performance difference (see Table 3).

We split our sub-analysis of GPT-3.5's answers into two groups: calculation and clinical questions. We then sorted them by their difficulty level and specific subtypes.

**Figure 2:**
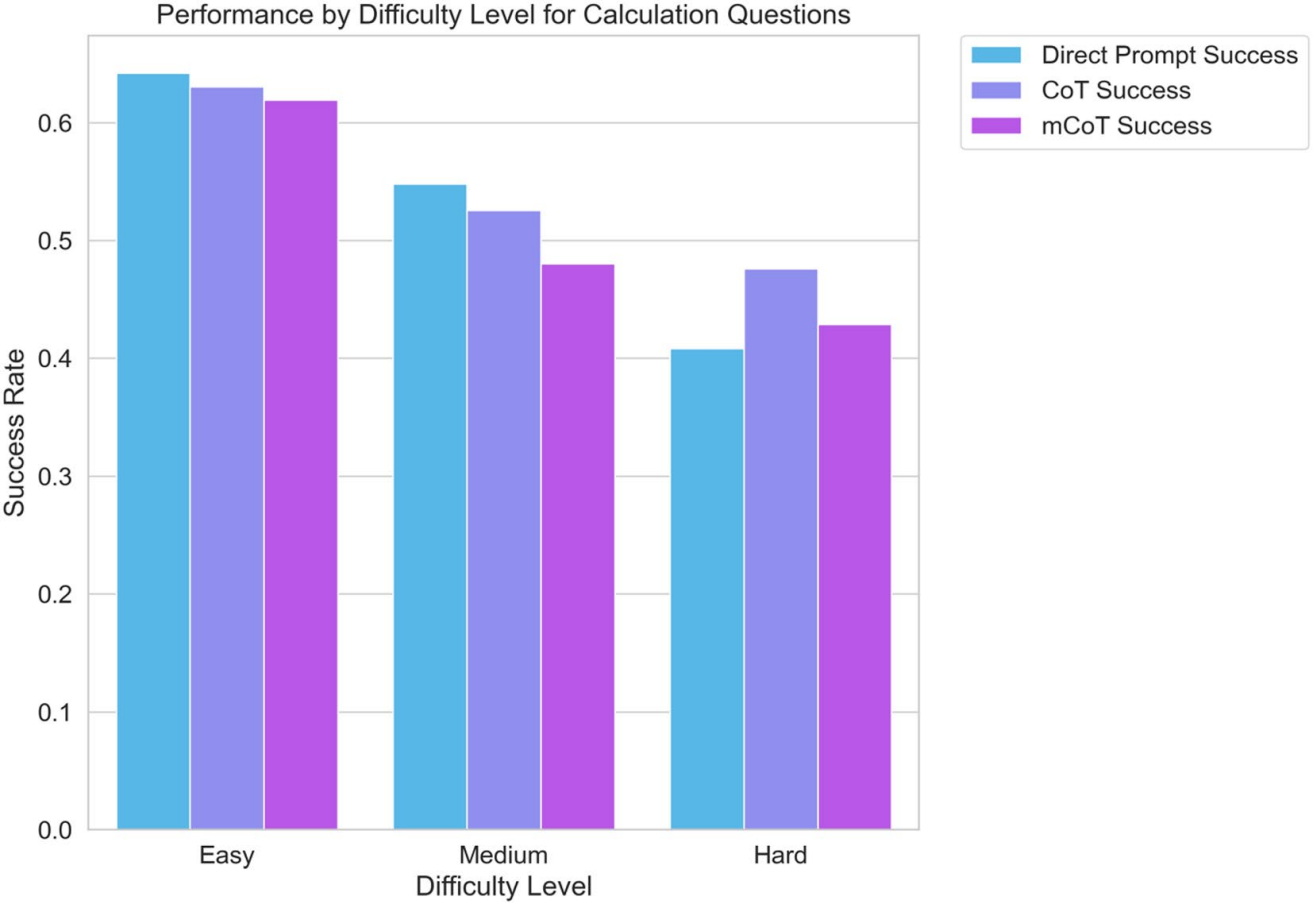
Bar graph representing the success rates of different prompting strategies ('Direct Prompt', 'CoT', and 'Modified CoT') for 'calculation' type questions across different difficulty levels ('Easy', 'Medium', 'Hard'). Each bar corresponds to the average success rate for the respective prompting strategy and difficulty level.

**Figure 3:**
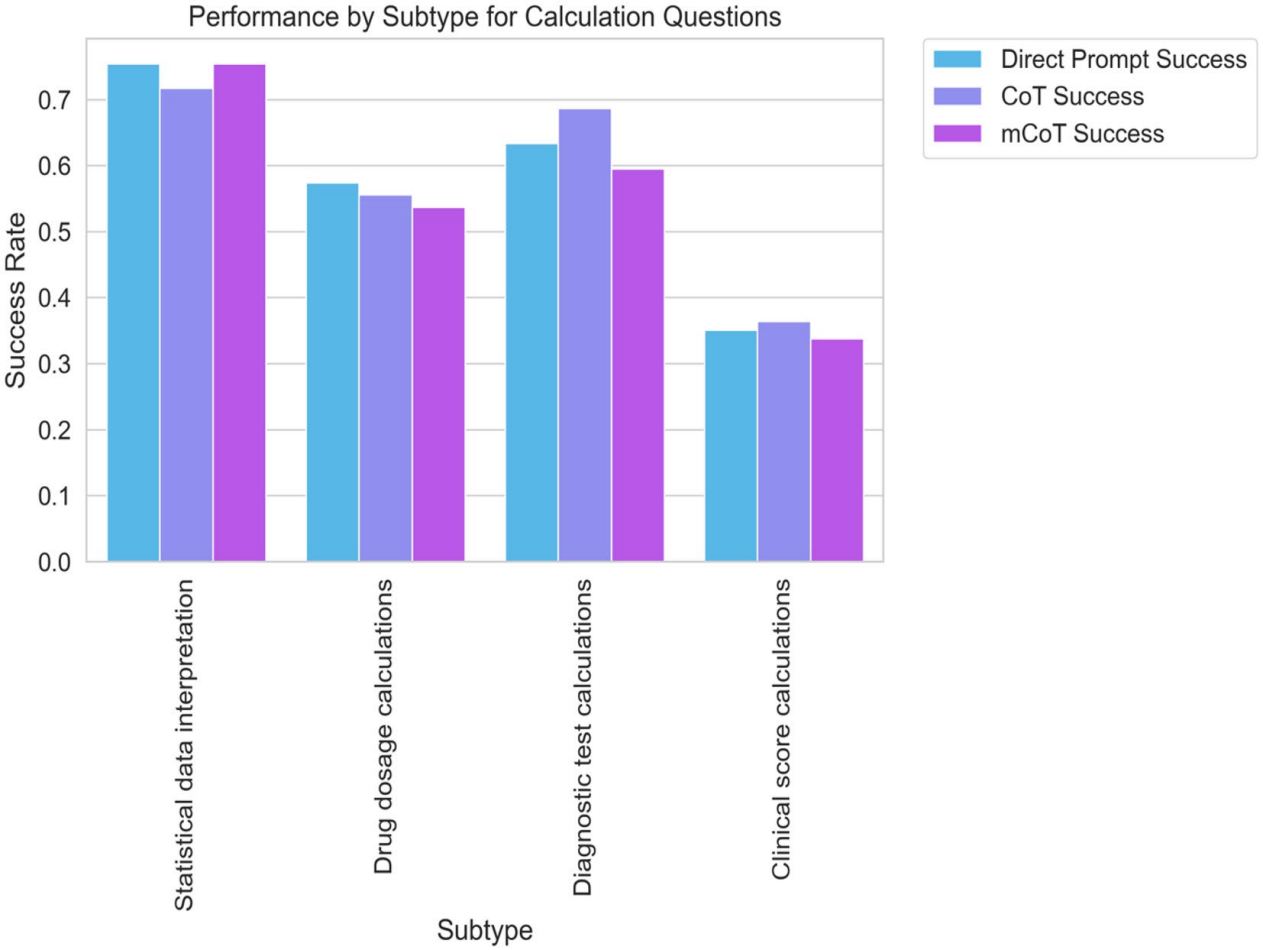
Bar graph showing the success rates of various prompting strategies ('Direct Prompt', 'CoT', and 'Modified CoT') for different 'calculation' question subtypes. Each bar represents the average success rate of a particular prompting strategy for a specific subtype.

For calculation-type questions, no one method of prompting was better than another, no matter the level of difficulty. For the easier questions, the three methods had similar accuracy: Direct Prompt had 64.2%, CoT had 63.1%, and Modified CoT had 61.9% (see Fig. 2). When we looked at the types of questions, no method stood out (refer to Fig. 3). The p-values were all above 0.05, showing no significant statistical differences.

**Figure 4:**
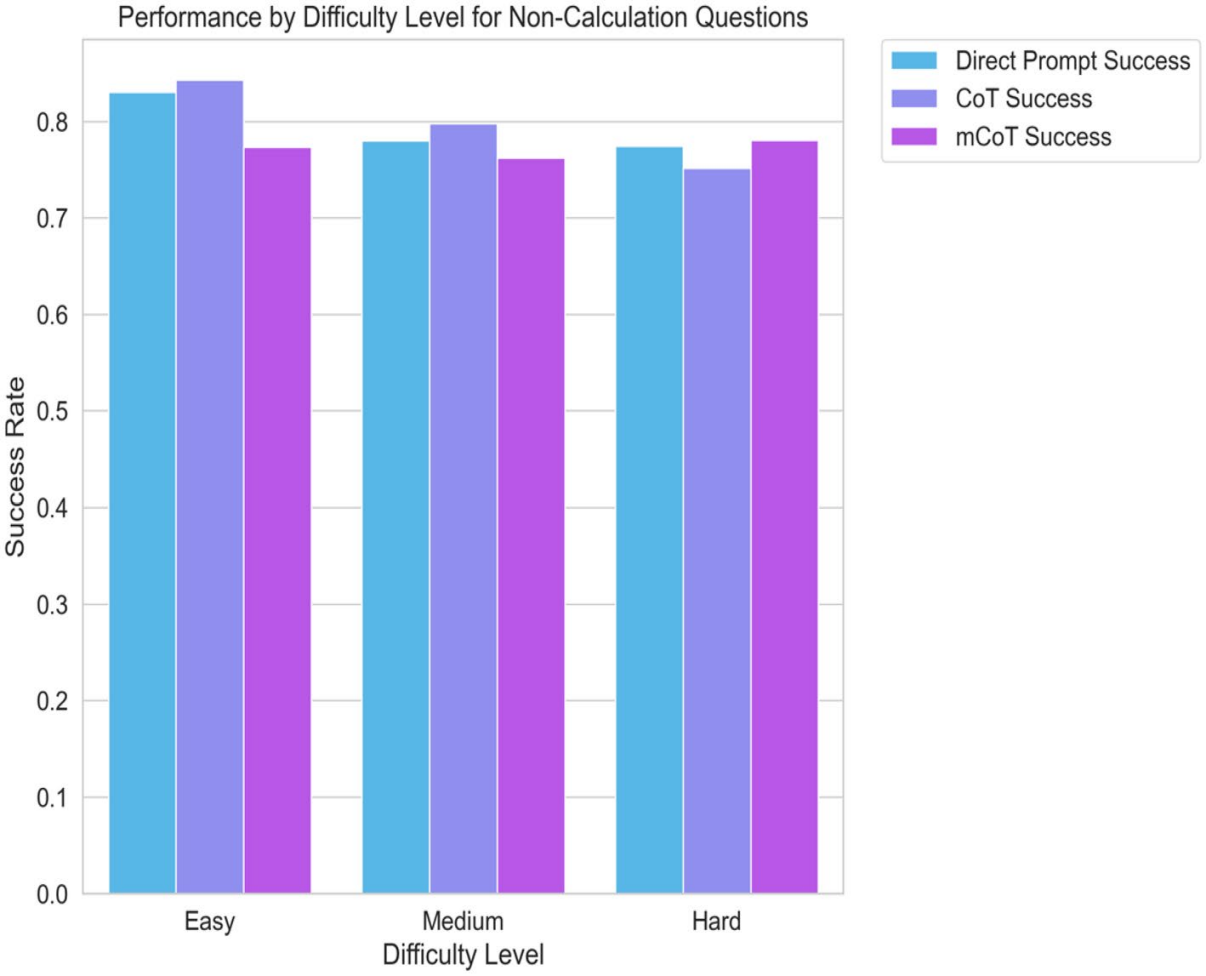
Bar graph depicting the success rates of the three prompting strategies ('Direct Prompt', 'CoT', and 'Modified CoT') for 'non-calculation' type questions across different difficulty levels ('Easy', 'Medium', 'Hard'). Each bar corresponds to the average success rate for a specific prompting strategy and difficulty level.

**Figure 5:**
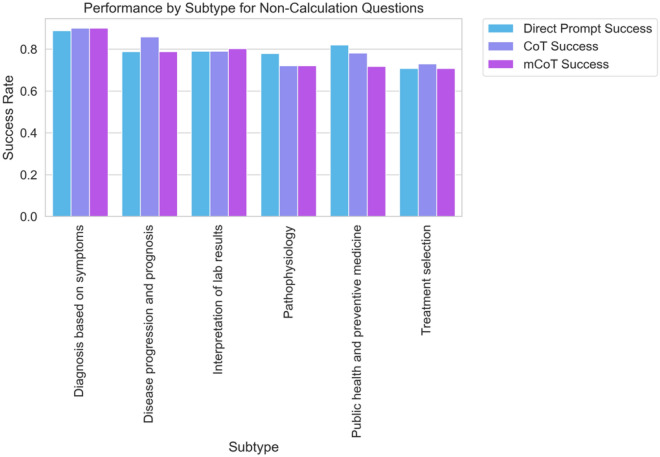
Bar graph illustrating the success rates of different prompting strategies ('Direct Prompt', 'CoT', and 'Modified CoT') for various 'non-calculation' question subtypes. Each bar represents the average success rate for a certain prompting strategy for a given subtype.

Similarly, in clinical questions, no method stood out. This was true for all levels of difficulty and subtypes of questions. For example, in diagnosing based on symptoms, success rates were close for all methods. Direct Prompt had 88.9%, CoT had 90.1%, and Modified CoT also had 90.1% (see Figs. 4 and 5). This pattern stayed the same in other areas, such as tracking disease progress, reading lab results, and picking treatments. The p-values showed no significant differences between the methods. Overall, our detailed analysis shows that while there might be small changes in how well different methods do in certain tasks, these are not large enough to be statistically important.

**Figure 6:**
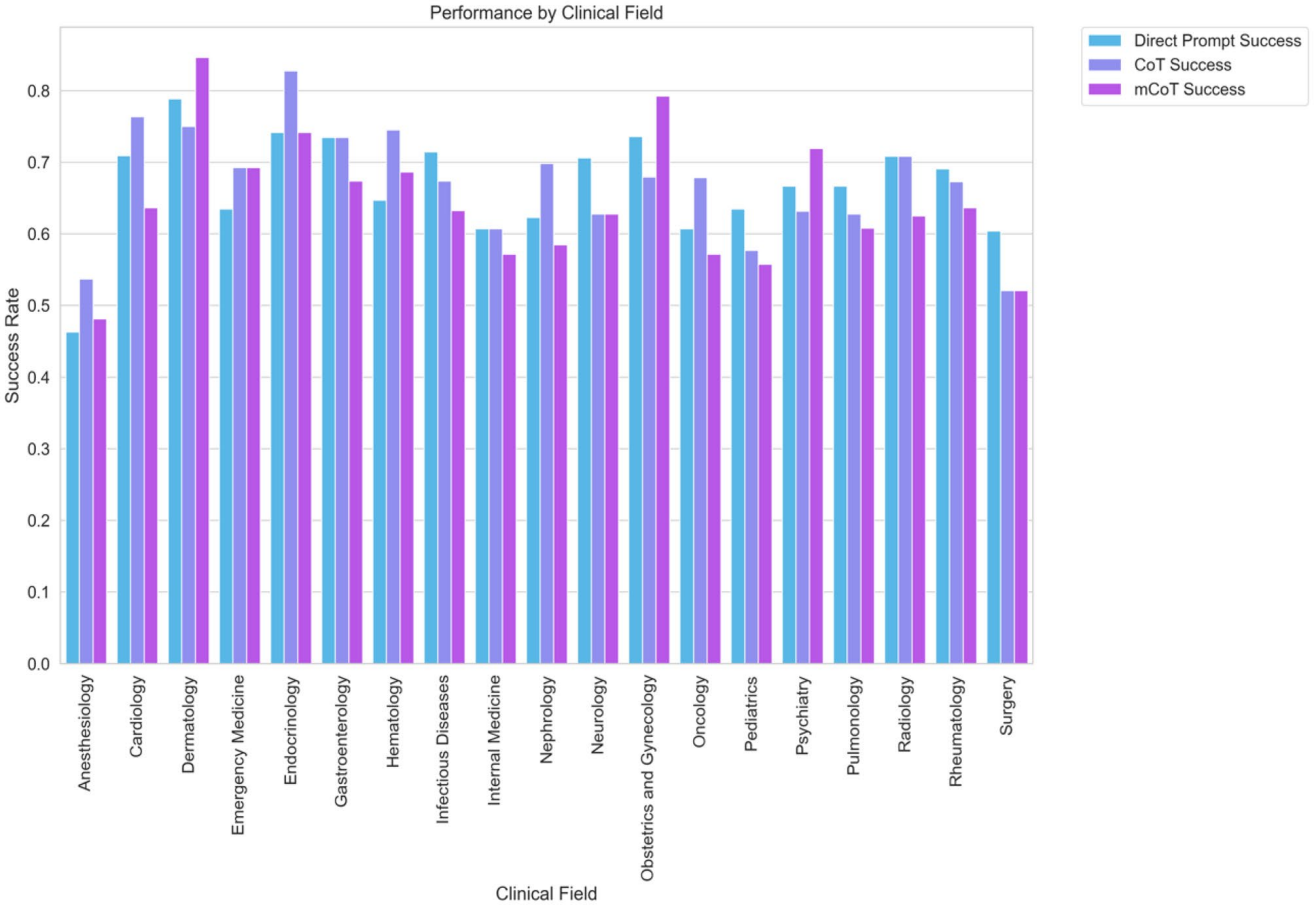
Bar graph detailing the success rates of the three prompting strategies ('Direct Prompt', 'CoT', and 'Modified CoT') across diverse medical fields. Each bar denotes the average success rate for a specific prompting strategy within a particular field.

We further looked at how GPT did in different medical fields (see Fig. 6). We again did not find a clear link to the kind of prompt used, but we did notice a clear overall pattern. Dermatology questions did the best with all methods, averaging around 79.5% success. In contrast, Anesthesiology questions did the worst, with an average success rate of about 49.4%.

## Discussion

Our study evaluated how well CoT prompt engineering works with ChatGPT for medical problems. We specifically separately evaluated calculation and clinical scenario question types. We tested three methods: direct prompt, the chain of thoughts (CoT), and a modified CoT. Surprisingly, we found no big differences in how they performed, even for medical calculation questions. All the methods did well in answering medical multiple-choice questions. We chose ChatGPT over GPT-4 for problem-solving. While GPT-4's power is clear, its high cost limits its use. The web interface of ChatGPT (GPT-3.5 model), on the other hand, is free. Also, GPT-3.5 API is much cheaper than GPT-4 API through OpenAI's API interface. GPT-3.5 API usage is around 30 times less costly than GPT-4^17^, making it a good choice for many uses.

Our study shows that prompt engineering for medical questions is complex^16^. The methods we used had similar results, but they are just a few among many. We did not look at other methods like self-consistency CoT^18^, which uses the most common answer from several CoT attempts, or "few-shot learning"^19^. Each method has its own strengths and weaknesses, and more research is needed to fully understand them.

A peculiar finding in our study was a discrepancy in accuracy across medical specialties. While the exact composition of GPTs' training data remains proprietary, leading to some uncertainty, it is plausible that the model has been exposed to dermatology content more extensively, potentially due to its broader applicability and the availability of visual and descriptive data, as opposed to the specialized and complex domain of anesthesiology.

The way OpenAI trains ChatGPT is mostly unknown. We do not know much about the training or inference strategies, but it is reasonable that they might use known prompt engineering methods. Our finding that different prompts worked the same might show this. ChatGPT might already use these methods in its training or during inference, making extra prompt methods like CoT unnecessary.

The growing complexity of LLMs such as ChatGPT points to a future where these tools are key in medicine. So, it is important to study and include them in medical training. Our study showed that a straightforward prompt worked as well as more complex ones. This makes it easier for healthcare workers to use these models. They do not need to learn complicated prompt techniques, which could lead to more adaptation of these tools in healthcare education and clinical settings.

Our study has limitations. First, we only used USMLE-style questions, so our results might not apply to other types of questions or fields. Second, we only looked at GPT-3.5 (ChatGPT). Other LLMs might respond differently to these prompt methods. Third, we didn't consider all multiple existing prompt engineering techniques and focused on CoT. Other techniques might affect the results. Fourth, by dividing questions into 'calculation' and 'non-calculation' types, we might have missed the complex mix often found in medical problem-solving. Fifth, the assignment of difficulty levels to questions via generative AI may not consistently correlate with expert evaluations or actual student performance, reflecting inherent subjectivity in perceived question complexity. Lastly, LLMs and the data sources that they are trained on advance rapidly and the results from this work may not generalize to future iterations of them.

In conclusion, CoT prompt engineering did not significantly change GPT-3.5's ability to handle USMLE-like medical calculations or clinical scenario questions. This suggests that ChatGPT's performance remains steady regardless of using the CoT technique. It could simplify AI's integration into medical education, allowing healthcare professionals to easily use tools like ChatGPT, without the need for intricate prompt engineering.

### Supplementary Information

Supplementary Information 1.

Supplementary Information 2.

Supplementary Information 3.

Supplementary Information 4.

Supplementary Information 5.

Supplementary Information 6.

## Data Availability

All data generated or analyzed during this study are included in this published article: Supplementary data: MAIN-Supp-prompt-engineering-answers.xlsx—Supplementary Information’s 1 and 2. Example of calculation and non-calculation questions: Calculation-nonCalculation_QuestionType_SupplimentFile.xlsx—Supplementary Information 3. Examples of Various Prompts Questions and Answers according to specific Clinical Field: Prompt_Examples_ClinicalFields.xlsx—Supplementary Information 5. Supplementary Data in SVPSS format: Main_Prompteng_supplementary_question_and_answer.sav—Supplementary Information 4.
